# [Corrigendum] Rapamycin enhances the sensitivity of ER-positive breast cancer cells to tamoxifen by upregulating p73 expression

**DOI:** 10.3892/or.2026.9139

**Published:** 2026-05-20

**Authors:** Lei Zhu, Xiao-Xia Li, Liang Shi, Jing Wu, Jia-Yi Qian, Tian-Song Xia, Wen-Bin Zhou, Xi Sun, Xu-Jie Zhou, Ji-Fu Wei, Qiang Ding

Oncol Rep 41: 455–464, 2019; DOI: 10.3892/or.2018.6842

Subsequently to the publication of the above paper, an interested reader drew to the authors' attention that, concerning the western blots shown in Fig. 1 on p. 457, the p73 data shown for the ZR75-1 and MCF-7 cell lines in Fig. 1A and B respectively were very similar if the latter blot were to be flipped vertically. The authors were able to re-examine their original data, and realized that the p73 data for the ZR75-1 cell line in Fig. 1A had inadvertently been copied across to show the results for the MCF-7 cell line experiments in Fig. 1B.

The revised version of Fig. 1, now showing the correct data for the p73 blot in Fig. 1B, is shown on the next page. Note that the correction made to this figure does not affect the overall conclusions reported in the paper. The authors are grateful to the Editor of *Oncology Reports* for allowing them the opportunity to publish this Corrigendum, and apologize to the readership for any inconvenience caused.

## Figures and Tables

**Figure 1.**
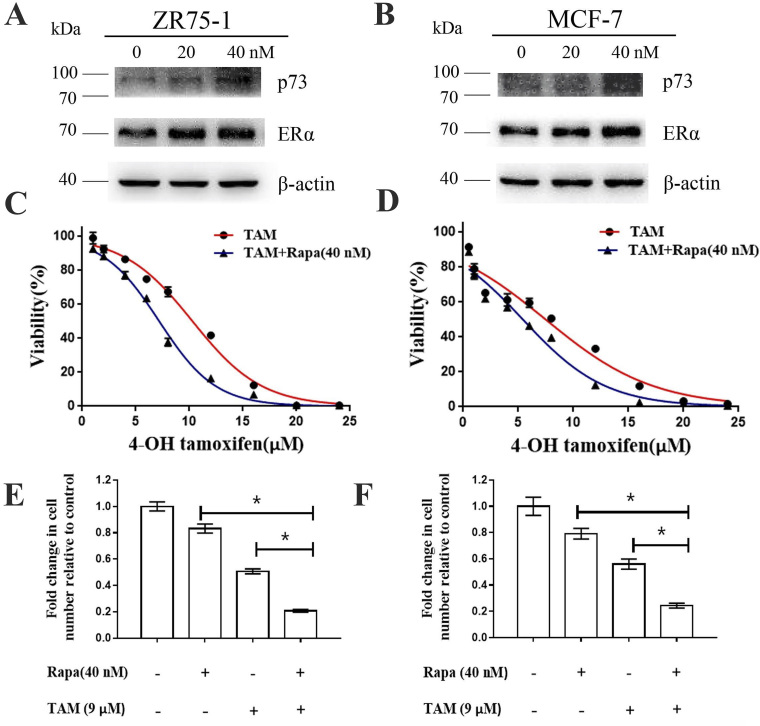
Rapamycin functions synergistically with tamoxifen in the MCF-7 and ZR-75-1 cells. (A and B) Changes in p73 and ERα expression following rapamycin treatment (C and D) MCF-7 and ZR-75-1 cells were treated with tamoxifen at various concentrations (0, 1, 2, 4, 6, 8, 16, 20 and 24 µM), or a combination of tamoxifen and rapamycin (40 nM). After 48 h, cell viability was measured by CCK-8 assays. (E and F) MCF-7 and ZR-75-1 cells were treated with 9 µM tamoxifen, 40 nM rapamycin, or a combination of the two agents for 48 h, and cell viability was measured. Histograms represent the quantification of cell viability. These data were calculated from 3 separate experiments and presented as the means ± SEM, *P<0.05 for the TAM (9 µM) group vs. the TAM (9 µM) + Rapa (40 nM) group. TAM, tamoxifen; Rapa, rapamycin.

